# Turning on virulence: Mechanisms that underpin the morphologic transition and pathogenicity of *Blastomyces*

**DOI:** 10.1080/21505594.2018.1449506

**Published:** 2018-08-01

**Authors:** Joseph A. McBride, Gregory M. Gauthier, Bruce S. Klein

**Affiliations:** a Division of Infectious Disease, Department of Medicine, University of Wisconsin School of Medicine and Public Health, 600 Highland Avenue, Madison, WI, USA; b Division of Infectious Disease, Department of Pediatrics, University of Wisconsin School of Medicine and Public Health, 1675 Highland Avenue, Madison, WI, USA; c Department of Medical Microbiology and Immunology, University of Wisconsin School of Medicine and Public Health, 1550 Linden Drive, Madison, WI, USA

**Keywords:** *Blastomyces*, Blastomycosis, BAD1, DRK1, DPPIVA, thermal dimorphism, phase transition, SREB

## Abstract

This review article focuses on the mechanisms underlying temperature adaptation and virulence of the etiologic agents of blastomycosis, *Blastomyces dermatitidis*, *Blastomyces gilchristii*, and *Blastomyces percursus*. In response to temperature, *Blastomyces* undergoes a reversible morphologic switch between hyphae and yeast known as the phase transition. The conversion to yeast for *Blastomyces* and related thermally dimorphic fungi is essential for virulence. In the yeast phase, *Blastomyces* upregulates the essential virulence factor, BAD1, which promotes attachment to host cells, impairs activation of immune cells, and blunts cytokine release. *Blastomyces* yeast also secrete dipeptidyl-peptidase IVA (DPPIVA), a serine protease that blunts the action of cytokines released from host immune cells. *In vivo* transcriptional profiling of *Blastomyces* yeast has uncovered genes such as *PRA1* and *ZRT1* involved in zinc scavenging that contribute to virulence during murine pulmonary infection. The discovery and characterization of genes important for virulence has led to advances at the bedside regarding novel diagnostics, vaccine development, and new targets for drug discovery.

## Introduction

*Blastomyces dermatitidis*, *Blastomyces gilchristii*, and *Blastomyces percursus* are the causal agents of blastomycosis [1,2]. *Blastomyces* species belong to a subset of pathogenic fungi within the Ascomycota phylum that include *Histoplasma capsulatum, Coccidioides immitis*, *Coccidioides posadasii*, *Paracoccidoides brasiliensis*, *Paracoccidioides lutzii*, *Sporothrix schenckii*, *Talaromyces marneffei* (formerly *Penicillium marneffei*), and *Emmonsia* species. These fungi are thermally dimorphic, which means they grow as filamentous mold in the environment and budding yeast in human tissue. The ability for the thermally dimorphic fungi to convert between hyphal and yeast forms is a fundamental part of their biology and is essential for pathogenesis. These fungi are capable of infecting humans and other mammals such as dogs and cats [1]. The majority of the dimorphic fungi can infect persons with both normal and impaired immune defenses [1]. This review of blastomycosis will highlight the link between morphologic switch and virulence as well as illustrate the innate and adaptive immune responses during infection.

## Mycology

*Blastomyces* species are haploid ascomycetes that undergo a reversible, temperature-dependent morphologic switch between hyphae at 22–25°C (ambient temperature) and yeast at 37°C (core human body temperature). *Blastomyces* yeast are multi-nucleate, have a doubly refractile cell wall when visualized by light microscopy, and divide by budding with mother and daughter cells separated by a broad-based bud [3]. The presence of broad-based budding yeast in a specimen (e.g., sputum, tissue) directly obtained from a patient is highly specific for *Blastomyces* infection. The yeast morphology among the 3 *Blastomyces* species is similar [2]. The mycelia of *B. dermatitidis* and *B. gilchristii* are characterized by septate hyphae with a single conidium attached to a conidiophore, similar to a lollipop [3]. In contrast, *B. percursus* has more elaborate conidiation structures including primary and secondary conidiophores bearing conidia [2]. In addition to phenotypic differences, the genome size of *B. percursus* (32.2 Mb) is smaller than *B. dermatitidis* (66.6 Mb) and *B. gilchristii* (75.4 Mb) [2]. Thus, the *B. percursus* genome is similar to other dimorphic fungi including *Histoplasma capsulatum, Coccidioides* species, and *Paracoccidioides* species [2]. Despite differences in genome size, the 3 *Blastomyces* species contain a similar number of genes [2]. The mechanism underlying the differences in genome size and how this may contribute to virulence is unknown.

## Geographic distribution and ecology

Blastomycosis is a predominantly North American disease. *Blastomyces* species are endemic to the Midwest, south-central, and southeast regions of the United States along the Ohio and Mississippi River valleys as well as U.S. states and Canadian provinces bordering the Great Lakes and St. Lawrence river [4]. Outbreaks of Blastomycosis associated with beaver dams and riverways within endemic areas have facilitated understanding of the specific ecological niche of *Blastomyces*, which is characterized by sandy soils with an acidic pH that are in forested areas with decaying vegetation and nearby fresh water [5,6]. Moreover, there are differences in the geographic distribution of *B. dermatitidis* and *B. gilchristii* in the endemic area. *B. dermatitidis* follows the traditionally accepted geographic range of blastomycosis from Canada to the southeastern United States. *B. gilchristii* is limited to Ontario, Saskatchewan, Alberta, Minnesota and Wisconsin [7].

Culture-proven cases of blastomycosis have been rarely reported outside of North America with approximately 100 cases from Africa and less than 10 cases from India [4]. Recently multilocus phylogenic analysis of *Blastomyces* species recovered from clinical specimens of three human cases outside North America (2 South Africa, 1 Israel) identified a new species, *B. percursus*, which is genetically distinct from *B. dermatitidis* and *B. gilcrhsitii* [2].

The majority of blastomycosis cases are sporadic; however, > 17 outbreaks have been reported since 1953 in the United States [4]. Activities that disrupt soil and aerosolize hyphal fragments or conidia can lead to infection. This includes construction of homes or roads, exploration of beaver dams, entering underground forts, forestry work, hunting, and water recreation (e.g., canoeing, tubing along a river) [4–6].

## The phase transition

The reversible morphologic transition between hyphae and yeast, known as the phase transition, defines the biology of the dimorphic fungi [8]. In the soil (22-25°C), these fungi grow as septate hyphae that produce conidia. Disruption of soil by human activities can aerosolize conidia and hyphal fragments. When inhaled into the lungs of a mammalian host (37°C), these infectious particles undergo a morphologic switch to pathogenic yeast (or spherules for *Coccidioides*) to cause pneumonia. The yeast phase facilitates evasion of host immune defenses and allows for extrapulmonary dissemination [4].

A shared biology allows for parallels and contrasts to be drawn between *Blastomyces* and other dimorphic fungi. Although temperature is the predominant stimulus for the phase transition, there are additional influences including carbon dioxide (CO_2_) tension, exogenous cysteine, and steroid hormones. Elevated CO_2_ tension (5% CO_2_) is required for *Coccidioides* arthroconidia to germinate into spherules and for optimal growth of *H. capsulatum* yeast in culture [9,10]. In *Blastomyces*, elevated CO_2_ is dispensable for germination of conidia and growth as yeast *in vitro*. In contrast, the uptake of exogenous cysteine by *Blastomyces* as well as by *H. capsulatum,* and *Paracoccidioides* is required to restart mitochondrial respiration, which transiently stops following a shift in temperature from 25^o^C to 37^o^C [11,12]. Without exogenous cysteine, the morphologic switch to yeast cannot be completed [11,12]. For some dimorphic fungi, the sex steroid hormone 17β-estradiol influences morphologic development and immune responses in women. The growth of *Coccidioides* spherules is accelerated following binding of 17β-estradiol, which may explain why pregnant women have an increased risk of disseminated coccidioidomycosis [13]. For *Paracoccidioides*, the conversion from conidia or hyphae to pathogenic yeast is blocked following binding of 17β-estradiol. The defect in the morphologic switch to yeast is associated with decreased transcription of genes involved in cell signaling, heat shock proteins, and cell wall remodeling [14–16]. The inhibitory effect of estradiol may account for the higher incidence of paracoccidioidomycosis in men compared to women [14]. In contrast, *Blastomyces* has limited capacity to bind estradiol [17].

## Morphologic conversion to the yeast phase promotes evasion of host immune defenses

The conversion to yeast is essential for *Blastomyces* and other dimorphic fungi to cause infection in mammalian hosts. Once inhaled into mammalian lungs, *Blastomyces* conidia are ingested by resident pulmonary macrophages and are capable of surviving intracellularly by converting to yeast and replicating [18]. *Blastomyces* yeast can also replicate extracellularly. This facultative intracellular lifestyle is not unique to *Blastomyces* and is important for the pathogenesis of *H. capsulatum, Coccidioides*, *Paracoccidioides*, *T. marneffei*, and *S. schenckii *[*19**-**23*]. For *H. capsulatum* and *Cryptococcus neoformans*, intracellular survival in macrophages promotes dissemination [24]. Whether *Blastomyces* uses a similar method for extrapulmonary dissemination is unknown. Approximately 25–40% of patients with blastomycosis have extrapulmonary dissemination, most often to the skin and bone [25].

During the phase transition to yeast, the thermally dimorphic fungi upregulate yeast phase-specific genes to actively subvert host immune defenses. *B. dermatitidis* and *B. gilchristii* increase the transcription of *BAD1* (*Blastomyces* adhesin-1; formerly WI-1), which encodes a 120kDA multifunctional, secreted protein that promotes tissue adhesion and immune evasion (Table 1) [26–31]. *In vivo* transcriptional profiling during murine pulmonary infection demonstrated that *BAD1* was the most upregulated gene in the trancriptome of *B. dermatitidis *[32]. Secreted BAD1 can remain soluble in the extracellular milieu or bind back to the yeast cell surface via interactions between the C-terminus of the BAD1 protein and chitin in the cell wall [27,29]. Cell surface-bound BAD1 promotes adhesion of yeast to host cells by binding heparin sulfate and complement receptors (CR3) [27,33]. In addition to facilitating adhesion, cell surface-bound BAD1 inhibits TNF-α production by macrophages and neutrophils in a transforming growth factor-β (TNF-β) dependent manner [30,31,33]. Soluble BAD1 also blocks TNF-α production via a mechanism that is independent of TGF-β [30]. Neutralization of TNF-α in mice results in more severe infection [31]. The Food and Drug Administration (FDA) published a warning that persons receiving TNF-α inhibitors are at increased risk for blastomycosis, histoplasmosis, and coccidioidomycosis [34]. BAD1 also impairs the activation of CD4^+^ T lymphocytes, and in turn, decreases their production of IL-17 and INF-γ [27]. BAD1 is essential for disease pathogenesis and deletion of the gene severely attenuates *Blastomyces* yeast during murine infection [26].

**Table 1.** Established and putative virulence factors in *Blastomyces*.

Gene	Function	Impact on virulence
*BAD1* (*Blastomyces* adhesin*-*1; formerly WI-1)	Promotes adhesion of yeast to host cells and immune evasion through inhibition of cytokines (TNF-α, IL-17 and INF-γ) and CD4^+^ T lymphocyte activation. *BAD1* is the most upregulated gene in *B. dermatitidis* during pulmonary infection.	*bad1*∆ yeast are attenuated during murine pulmonary infection. When injected subcutaneously in mice, *bad1*∆ yeast induce sterilizing immunity against lethal pulmonary infection.
*DPPIVA* (dipeptidyl-peptidase)	Serine protease that degrades GM-CSF and impedes recruitment of innate immune cells	*DPPIVA* knockdown strains have reduced survival in the presence of activated innate immune cells and attenuated virulence in murine pulmonary infection
Cysteine synthase	Involved in the biosynthesis of L-cysteine.	Important for transition to yeast and maintenance of the yeast-phase growth.
*CDG* (cysteine dioxygenase)	Catabolic enzyme that catabolizes L-cysteine to L-cysteine sulfonic acid, which can further be broken down to sulfite.	In *C. albicans* deletion of *CDG1* attenuates virulence during murine infection. *Arthroderma benhamiae cdg*∆ mutants have reduced growth on hair and nails. Potential to contribute to growth of *Blastomyces* on keratinized structures.
*SSU1*	Transmembrane sulfite efflux pump. Sulfite is toxic to cells and is secreted via the SSU1 efflux pump.	*Arthroderma benhamiae ssu1*∆ mutants have impaired growth on hair and nails. Potential to contribute to growth of *Blastomyces* on keratinized structures.
*PRA1, ZRT1*, *ZRT2*	*PRA1* encodes a zincophore. *ZRT1* and *ZRT2* encode high and low affinity zinc transporters, respectively. These genes are upregulated in yeast during pulmonary infection.	*C. albicans pra1*∆ mutants have decreased ability to lyse endothelial cells under zinc-poor conditions. *Blastomyces pra1*∆ and *zrt1*∆ mutants are impaired in establishing infection in mice.
*NIC1*	Transports nickel which is used as a cofactor for urease (Urea → Ammonia + CO_2_).	In *C. neoformans*, deletion of *NIC1* results in decreased ability of yeast cells to enter CNS. Potential to contribute to *Blastomyces* virulence or dissemination.
*DRK1*	Essential for the temperature-dependent conversion to yeast at 37^o^C.	*DRK1* silenced strains of *B. dermatitidis* and *H. capsulatum* are avirulent in a murine model of pulmonary infection. These strains also exhibit defects in cell wall integrity and fail to upregulate yeast-phase specific virulence factors (*BAD1*, *CBP1*). *T. marneffei drkA*∆ mutants have impaired conidial germination in macrophages.
*RYP1-4* (Required for yeast phase 1-4)	Conserved transcription factors in the dimorphic fungi that are essential for the morphologic switch to yeast at 37^o^C	RNAi-silenced *RYP1-4 H. capsulatum* strains fail to convert to yeast at 37^o^C and are unable to upregulate yeast-phase specific virulence genes (*CBP1*, *YPS3*).

In addition to BAD1, the secretion of a serine protease known as dipeptidyl-peptidase IVA (DPPIVA) contributes to virulence *in vivo* (Table 1) [35]. *B. dermatitidis DPPIVA* silenced strains have reduced survival in the presence of activated innate immune cells and attenuated virulence in a murine model of pulmonary infection [35]. DPPIVA degrades granulocyte-macrophage colony stimulating factor (GM-CSF), a cytokine that activates neutrophils and macrophages to control fungal infection *in vivo* [35]. DPPIVA also impedes the recruitment of innate immune cells [35].

During phase transition, changes in cell wall carbohydrate composition may also contribute to virulence and immune evasion. During the conversion from mold to yeast, the content of cell wall α-(1,3)-glucan increases, whereas β-(1,3)-glucan decreases from 40% to less than 5% [36]. The reduction of β-(1,3)-glucan in the yeast cell wall may limit dectin-1-mediated recognition by host immune cells and mannose-binding lectins [37].

Although *Blastomyces* uses multiple strategies to subvert immune defenses to establish infection in healthy persons, these mechanisms are not infallible. The host can mount an immune response to halt the progression of infection. On the basis of outbreak investigations, an estimated 50% of persons exposed to *Blastomyces* develop symptomatic infection, most commonly pneumonia [5,6]. The remaining 50% of exposed persons will experience asymptomatic or subclinical infection [5,6].

## Identification of genes via *in vivo* transcriptional profiling that have the potential to influence virulence in *Blastomyces*

Traditionally, novel virulence factors have been discovered using forward and reverse genetic approaches *in vitro*. The development of high-throughput RNA sequencing along with the ability to quickly isolate *Blastomyces* yeast from lung tissue has led to the ability to perform *in vivo* transcriptional profiling of yeast during murine pulmonary infection [32,38]. To uncover genes potentially important for pathogenesis and virulence, RNA seq analysis was used to compare *B. dermatitidis* yeast harvested from murine lung tissue versus yeast grown *in vitro* at 37^o^C (with and without macrophages) and hyphae at 22°C [32]. K-means cluster analysis identified 72 genes that were upregulated >2-fold during pulmonary infection and independent of temperature, co-cultivation with macrophages, and media conditions [32]. Upregulated genes of interest include those involved in amino acid metabolism and metal uptake (Table 1). In this gene set, the two most highly upregulated genes were involved in cysteine metabolism (cysteine synthase) and exogenous zinc uptake (*PRA1*) [32]. Recent molecular developments exploiting CRISPR/Cas9 for high frequency gene targeting and editing in *Blastomyces* have revealed that the secreted zincophore PRA1 and its transporter ZRT1 are required for disease pathogenesis in a murine model of infection (Table 1) [39].

Cysteine synthase is involved in biosynthesis of L-cysteine from acetyl-L-serine. As previously mentioned, cysteine is important for the phase transition and maintenance of the yeast phase. In addition to cysteine synthase, other genes involving cysteine metabolism were upregulated including cysteine dioxygenase (*CDG*) and a transmembrane sulfite efflux pump (*SSU1*) [32]. *CDG* encodes a catabolic enzyme that converts L-cysteine to L-cysteine sulfonic acid, which can be further broken down to pyruvate and sulfite. Sulfite accumulation is toxic to cells and is secreted into the extracellular milieu via the Ssu1 efflux pump. In *C. albicans*, deletion of *CDG1* null mutants (*cdg1*Δ) attenuates virulence during murine infection [40]. In *Arthoderma benhamiae*, a dermatophyte, the catabolism of cysteine to sulfite and its efflux promotes fungal growth on keratinized structures [41]. Sulfite breaks down keratin by cleaving disulfide bonds [41]. Cysteine dioxygenase and *SSU1* null mutants exhibit reduced growth on hair and nails [41]. There is potential that cysteine biosynthesis and catabolism followed by sulfite secretion could promote the growth of *Blastomyces* yeast in skin, the most common site for extrapulmonary dissemination.

During *in vivo* infection, *B. dermatitidis* yeast upregulate genes important for exogenous zinc uptake including a zincophore encoded by *PRA1*, a high-affinity zinc transporter *ZRT1*, and a low affinity zinc transporter *ZRT2* [32]. In *C. albicans*, the PRA1 zincophore is secreted in the extracellular environment, binds zinc, and brings it back to it to the fungal cell via its interaction with ZRT1 at the plasma membrane [42]. In addition to zinc uptake, *B. dermatitidis* increases the transcription of a nickel transporter, *NIC1* [32]. Nickel serves as a cofactor for urease, which catalyzes the conversion of urea to ammonia and CO_2_. In mammalian tissues, catabolism of purine nucleotides results in the production of urea. In *C. posadasii*, deletion of the urease gene attenuates virulence of the *ure*Δ mutant in murine model of pulmonary infection [43]. Similarly, deletion of *NIC1* and *URE1* in *C. neoformans* results in decreased ability of yeast cells to enter the central nervous system [44].

## Molecular regulation of the phase transition

The development of molecular tools to dissect the pathogenesis of the thermally dimorphic fungi has led to the discovery of genes and gene networks that regulate the phase transition. The discovery of *DRK1*, which encodes an intracellular group III hybrid histidine kinase, demonstrated that the morphologic switch to yeast at 37^o^C is required for virulence of *Blastomyces* and *Histoplasma* (Table 1) [45,46]. *DRK1* insertional mutants, null mutants (*drk1*∆), and RNA interference (RNAi) strains fail to convert to yeast, remain constitutively hyphal at 37^o^C, do not upregulate important yeast-phase specific virulence genes including *Blastomyces BAD1* and *Histoplasma CBP1* (calcium-binding protein-1), and display defects in cell wall integrity [45]. *DRK1*-silenced strains of *Blastomyces* and *Histoplasma* are avirulent in a murine model pulmonary infection following intratracheal instillation of conidia [45]. The deletion mutants were unable to be tested *in vivo* because *drk1*∆ strains produce few conidia [45]. The function of *DRK1* is conserved in other thermally dimorphic fungi. Deletion of *drkA*, a homolog of *DRK1* in *T. marneffei*, impairs the germination of conidia to yeast during co-cultivation with macrophages [47]. Transcriptional analysis has demonstrated upregulation of *drkA* in *T. marneffei* yeast at 37^o^C. Similarly, *DRK1* transcript is upregulated in yeast (versus hyphae) in *Paracoccidioides* and *Sporothrix schenckii* [48,49]. *DRK1* is postulated to participate in the high-osmolarity glycerol (*HOG1*) signaling pathway. The *HOG1* cascade facilitates adaptation to a variety of exogenous stimuli including temperature, oxidative stress, and osmotic stress. Under osmotic stress*, DRK1* and *drkA* transcription is upregulated in *Paracoccidioides* and *T. marneffei*, respectively [47,49].

In addition to *DRK1*, *RYP1-4* (required for yeast phase) transcription factors regulate the morphologic switch and influence the transcription of yeast-phase specific genes at 37°C in *H. capsulatum* (Table 1) [50–52]. *RYP1-4* transcription factors are conserved in filamentous and dimorphic fungi including *Blastomyces*. *RYP1* – *RYP3* are homologs of *WOR1, VosA,* and *VelB* respectively [50,51]. WOR1 functions as a master regulator of white-opaque or white-gray-opaque switching in *C. albicans, C. tropicalis,* and *C. dubliniensis* [53,54]. *RYP2* and *RYP3* are homologs of *VosA* and *VelB*, which are components of the velvet complex (VosA-VelB-LaeA). In filamentous fungi, the velvet complex coordinates fungal development and production of secondary metabolites [55]. *RYP4* encodes a Zn(II)_2_Cys_6_ zinc binuclear cluster domain protein [52]. In *H. capsulatum*, RYP1-4 transcription factors are part of an integrated network that directly regulates the transcription of a common set of genes including those important for virulence such as *CBP1* and *YPS3* (yeast-phase specific-3) [52]. CBP1 induces macrophage apoptosis, whereas YPS3 promotes extrapulmonary dissemination [56,57]. RNAi-silenced *RYP1-4* strains fail to undergo the morphologic switch and grow as hyphae at 37°C [50–52].

The temperature-dependent transition in the opposite direction, yeast to hyphae, is governed by a GATA transcription factored encoded by *SREB* in *B. dermatitidis*, and its homolog, *SRE1*, in *H. capsulatum* [58–60]. *SREB* deletion mutants (*sreb*Δ) and *SRE1*-knockdown strains are unable to complete the conversion from yeast to hyphae following a drop-in temperature from 37^o^C to 22–25^o^C [58–60]. In *B. dermatitidis sreb*∆ strains, the morphologic defect at 22^o^C is temporally associated with the reduction in neutral lipid (ergosterol, triacylglycerol) biosynthesis and loss of lipid droplets [59]. The defects in phase transition and lipid droplet formation are partially restored by supplementing the media with exogenous saturated fatty acids [59]. In contrast, unsaturated fatty acids do not correct the defects in filamentous development and lipid droplets [59]. Collectively, these data suggest that neutral lipid metabolism contributes to filamentous development at ambient temperature in *Blastomyces* [59]. In contrast, the role of *SREB* (and *SRE1*) as a negative regulator of genes involved in iron assimilation appears to be independent of phase transition [59,60]. Nevertheless, iron has the potential to influence morphogenesis. Deletion of *VMA1*, which encodes a vacuolar ATPase important for iron homeostasis, renders *H. capsulatum* yeast incapable of converting to hyphae at room temperature [61]. However, the mechanism underlying the defect in the phase transition due to loss of *VMA1* has yet to be characterized. In *T. marneffei*, hyphal development and maintenance of filamentous morphology is regulated by transcription factors encoded by *hygr*A and *tupA*, respectively [62,63].

Similar to *C. albicans*, exposure of *Blastomyces* and *Histoplasma* to N-acetylglucosamine (GlcNAc) accelerates the conversion from yeast to hyphae at ambient temperature and promotes hyphal growth [64]. This event is mediated by *NGT1* and *NGT2*, which encode N-acetylglucosamine transporters 1 and 2, respectively [64]. Both of these transporters are functional and RNAi interference of *NGT1* or *NGT2* in *H. capsulatum* impairs the conversion to mycelia at room temperature [64]. The effect of GlcNAc on the phase transition is specific and does not occur with other carbohydrates such as fructose or glucosamine [64]. Moreover, GlcNAc does not alter the morphology or growth rate of yeast [64]. How GlcNAc induces temperature-mediated hyphal development is unknown; however, it is likely independent of GlcNAc metabolism because RNA silencing of *HXK1*, which encodes a kinase important for GlcNAc catabolism and glycan biosynthesis, does not alter the morphologic switch [64]. Whether NGT1 and NGT2 directly sense GlcNAc or import it for intracellular sensing remains to be determined [64].

The phase transition from *Blastomyces* yeast to hyphae following a drop-in temperature to 22–25^o^C is important and contributes to pathogenesis. The hyphal form is critical for the production of conidia, which allows for thermally dimorphic fungi in soil to be transmitted to mammalian hosts. Yeast (and spherules) are not transmissible via the airborne or respiratory routes. Similarly, hyphal growth is essential for mating, which promotes genetic diversity in *Blastomyces* as well as other fungi [65,66]. *Blastomyces* mating occurs exclusively in the mycelial form when hyphal filaments of opposite mating types – MAT1-1 and MAT1-2 – join together to exchange genetic material [65]. Both mating types are capable of causing infection.

## Host response

The host response to *Blastomyces* infection requires both innate and adaptive immune defenses. Although *B. dermatitidis* can germinate and replicate intracellularly, alveolar neutrophils and macrophages are capable of killing a large percentage of conidia [67]. Conidia that survive the initial host response can germinate to yeast, which are more challenging to kill. Yeast are relatively resistant to reactive oxygen species, do not induce an intense oxidative response, and are capable of suppressing macrophage nitric oxide production [68]. The adaptive immune response involves T-helper 17 and T-helper 1 cells releasing cytokines such as IL-17, TNF-α, and INF-γ to promote macrophage fungicidal activity [69,70]. Following acute blastomycosis, cell-mediated immunity persists for at least 2 years and possibly longer [71]. This finding suggests that after recovery from blastomycosis, there is potential for protective immunity against re-infection; however, clinical studies in humans are lacking with regard to long-term protective immunity after infection.

## Bench-to-bedside advances

Research discoveries over the past 2 decades have begun to influence the diagnosis of blastomycosis. Traditional serologic testing by immunodiffusion and complement fixation for the diagnosis of blastomycosis suffers from low sensitivity (≤ 28%) [72]. A new serologic assay to detect antibodies against BAD1 has enhanced sensitivity (87%) compared to the traditional assays and is highly specific (94-99%) [73]. Because BAD1 is unique to *Blastomyces*, the BAD1 assay can distinguish between blastomycosis and histoplasmosis [73]. Cross-reaction between existing antibody and antigen tests for *Blastomyces* and *Histoplasma* is common. Similarly, a real-time PCR assay that amplifies the *BAD1* promoter has been developed. This is a highly specific diagnostic test that allows for rapid identification of *B. dermatitidis* in culture and in paraffin-embedded tissues [74]. The ability of PCR to identify *Blastomyces* in paraffin-embedded tissues can facilitate the diagnosis when there is a paucity of yeast in the sample or a lack of characteristic broad-based budding [74].

Although β-(1,3)-glucan assays and echinocandin antifungal drugs have respectively improved the diagnosis and treatment of many fungal infections, the low level of β-(1,3)-glucan in the *Blastomyces* yeast cell wall precludes the use of β-(1,3)-glucan assays for accurate diagnosis and renders the echinocandin antifungal drug class ineffective [4,25]. Therefore, effective treatment of blastomycosis is limited to the azole antifungals and polyene amphotericin B formulations [25]. Thus, the development of novel antifungal drugs is important. The group III hybrid histidine kinase is being investigated as a target for antifungal drug discovery [75,76]. Similarly, an attenuated *B. dermatitidis* yeast strain devoid of BAD1 (*bad1*∆) is being used to uncover mechanisms important for fungal vaccine development. Mice vaccinated with *bad1*∆ yeast develop sterilizing immunity following a lethal experimental challenge with *B. dermatitidis* [77]. Research on *bad1*∆ vaccine-induced immunity has led to the discovery that calnexin has a conserved sequence among pathogenic ascomycetes that makes it a potential pan-fungal vaccine target [78], and that *Blastomyces* endoglucanase-2 (Bl-Eng2) represents a novel ligand for dectin-2 with potent adjuvant properties for the induction of Th17 immune cells [79].

## Conclusion

*Blastomyces* species are ascomycete pathogens that are capable of infecting both immunocompromised and the immunocompetent persons. The phase transition from mycelia-to-yeast is essential for virulence. This morphologic switch results in the upregulation of virulence factors that promote adhesion to host tissues, growth in macrophages, dysregulation of the host cytokine response, and blunts the effectiveness of cell-mediated immune defenses. Modern molecular techniques have provided novel insight into the phylogeny and pathogenesis of *Blastomyces*. The discovery of virulence factors such as BAD1 and DRK1 have led to new diagnostic tests and formed a foundation for the development of new antifungal drugs and experimental vaccines.
